# Revealing Prognosis-Related Pathways at the Individual Level by a Comprehensive Analysis of Different Cancer Transcription Data

**DOI:** 10.3390/genes11111281

**Published:** 2020-10-29

**Authors:** Jingya Fang, Cong Pian, Mingmin Xu, Lingpeng Kong, Zutan Li, Jinwen Ji, Liangyun Zhang, Yuanyuan Chen

**Affiliations:** 1 College of Agriculture, Nanjing Agricultural University, Nanjing 210095, China; 2018201002@njau.edu.cn (J.F.); 2016201007@njau.edu.cn (M.X.); 2017201005@njau.edu.cn (L.K.); 2019201003@njau.edu.cn (Z.L.); 2020201011@stu.njau.edu.cn (J.J.); 2 Department of Mathematics, College of Science, Nanjing Agricultural University, Nanjing 210095, China; piancong@njau.edu.cn

**Keywords:** TCGA, prognosis-related pathways, cancer, iPS

## Abstract

Identifying perturbed pathways at an individual level is important to discover the causes of cancer and develop individualized custom therapeutic strategies. Though prognostic gene lists have had success in prognosis prediction, using single genes that are related to the relevant system or specific network cannot fully reveal the process of tumorigenesis. We hypothesize that in individual samples, the disruption of transcription homeostasis can influence the occurrence, development, and metastasis of tumors and has implications for patient survival outcomes. Here, we introduced the individual-level pathway score, which can measure the correlation perturbation of the pathways in a single sample well. We applied this method to the expression data of 16 different cancer types from The Cancer Genome Atlas (TCGA) database. Our results indicate that different cancer types as well as their tumor-adjacent tissues can be clearly distinguished by the individual-level pathway score. Additionally, we found that there was strong heterogeneity among different cancer types and the percentage of perturbed pathways as well as the perturbation proportions of tumor samples in each pathway were significantly different. Finally, the prognosis-related pathways of different cancer types were obtained by survival analysis. We demonstrated that the individual-level pathway score (iPS) is capable of classifying cancer types and identifying some key prognosis-related pathways.

## 1. Introduction

With the rapid development of next-generation sequencing technologies and the decreasing costs of high-throughput sequencing, information on disease mutations associated with different types of cancer has been used in clinical research and clinical practice [1,2]. To obtain information about cancer treatment and progression, genome sequencing is already being used to aid diagnoses in early efforts to construct predictive models of disease for healthy individuals [3,4,5]. Using whole-genome sequencing, whole-exome sequencing, and RNA sequencing, we can obtain the genomes and transcriptomes of cancer patients [6,7,8,9,10,11,12,13,14]. For example, The Cancer Genome Atlas (TCGA) program, which contains multiple platforms and different types of data for 33 cancer types, such as gene expression, protein expression, mutation, DNA methylation, and copy number variation data [15], has been used to analyze problems regarding disease. By comparing tumor and non-tumor data, cancer-specific tumorigenic features can be discovered [7,8,9]. In particular, omics data can reveal what is taking place in disease at the molecular level, enabling diseases to be related through disease-associated genes [16,17,18], gene expression [19,20,21], protein interaction networks [22], pathways [23], and biological processes [24]. Molecular signatures, which are obtained from high-throughput expression data, have long been appreciated and discussed in precision medicine for cancer. Some prognostic genes can classify cancer patients into different risk subgroups with distinct outcomes by high-throughput expression data [25]. Several commercialized gene signatures have been approved for risk prediction in clinical practice, such as PAM50 for breast cancer [26,27,28], Mammaprint^®^ [27], a set of 70 genes for low- or high-risk prediction in breast cancer, and OncoType DX^®^ panels for tumor profiling in breast [28], prostate [29], and colon [30] cancers.

Although prognostic gene lists have had success in prognosis prediction, they are strongly depended on the selection of patients in the training sets [31]. In fact, it has been recognized that using single genes that are related to the relevant system or specific network cannot fully reveal the phenotypic change of a living organism. Thus, researchers proposed pathway and network analyses, which can reduce data involving many altered genes and proteins to a smaller and more interpretable set of altered processes [32,33]. By pathway and network analyses, driver genes and pathways in different cancer types [34,35] can be found, new cancer mechanisms and biomarkers can be proposed [36], and key regulators of cancer-related gene networks can also be identified [37,38]. Currently, molecular networks have been widely applied to human diseases. For example, some network-based methods can identify disease modules and pathways as well as clarify molecular mechanisms of disease development at the network level [39,40,41]. Biomarkers based on networks have been proposed, such as subnetwork markers [39,40], network biomarkers [42], and edge biomarkers [43,44]. Compared with traditional single-molecule biomarkers, these network-based biomarkers characterize disease states more accurately because more information on interactions and networks is considered. Moreover, some systematic pathway-based approaches, which include information about the molecular mechanisms among individual genes, proteins, or metabolites, can extract more stable and interpretable features for risk prediction. Predefined pathways can be obtained from biological databases such as Kyoto Encyclopedia of Genes and Genomes (KEGG) [45] and Reactome [46]. Cancer is characterized by tremendous phenotypic heterogeneity; thus, a unique plan for individual tumor samples to identify the most promising treatment strategy for the patient is crucial and is also in accordance with the target of new precision medicine. Because of the heterogeneity of cancer, identifying biomarkers that can predict clinical progression and outcomes will be useful. For example, Uhlen et al. [47] found some prognostic genes whose expression level strongly correlated with the patients’ overall survival. Some previous methods analyzed single samples based on networks or pathways [48,49]. Drier et al. proposed the personal pathway deregulation score (PDS), which is based on the principal curve and then calculates the distance between a single tumor sample and the median of normal samples. They applied principal component analysis to reduce the dimensions to obtain the best principal curve. However, extracting the principal curve to interpret a single sample was not accompanied by a cohort dataset. Liu and Wang et al. [50] developed a statistical method, the sample-specific network (SSN) method, based on the molecular expression of a single sample to construct individual-specific networks. They first proposed a method to predict driver genes based only on the expression profile of a single sample.

Because most of the existing pathway-based studies [51,52,53] have focused on groups of tumors or normal samples, we intended to develop a novel method that can calculate the perturbation of a pathway with reference to normal samples at the individual level.

In this study, we developed a novel method to calculate the individual-level pathway score (iPS) of a single sample rather than aggregating a group of samples. This method converts gene-level information into pathway-level information at the individual level, which can characterize a single sample in a biological system. Then, all cancer samples can be divided into perturbed-weak and perturbed-strong samples based on the degree of iPS deviation from that of normal samples. Furthermore, iPS was used to identify prognosis-related pathways, which could significantly distinguish perturbed-weak and perturbed-strong samples. To demonstrate that iPS can sensitively capture relevant biological and clinical information, we applied it in a pan-cancer analysis using 16 cancer types in The Cancer Genome Atlas (TCGA) database based on the pathways in the Reactome database [54]. Our results showed that iPS can distinguish different cancer types well. The prognosis-related pathways of the 16 cancer types have excellent ability to distinguish perturbed-weak and perturbed-strong samples. Thus, we trust that our pathway-based method is reliable in prognostic prediction, especially for precision medicine.

## 2. Materials and Methods

### 2.1. Data Preparation

Normalized RNA-seq data were downloaded from The Cancer Genome Atlas (TCGA) data portal by using TCGA-Assembler 2 (v2.0.6, http://www.compgenome.org/TCGA-Assembler/). In total, 16 cancer types and their tumor-adjacent normal tissue were studied, including 6548 tumor samples and 688 normal samples. The genes with zero expression values in more than 75% of all samples in each cancer type were excluded from the following analysis. Out of all 20,502 genes, 3299 genes were excluded. Finally, we obtained 17,203 genes. Additional clinical data of all samples were downloaded using the R package RTCGA. The specific information of the data sets from TCGA used in this study is summarized in Table 1.

Pathway information was obtained from the Reactome Pathway Database [54]. The ReactomeFIPlugIn app can help us access the Reactome Functional Interaction network, a highly reliable, manually curated pathway-based network that extends curated pathways with non-curated sources of information, including protein-protein interactions, gene co-expression, protein domain interaction, Gene Ontology [55] annotations, and text-mined protein interactions [56]. We downloaded all the Reactome pathways (216 in total) by using the ReactomeFIPlugIn app in Cytoscape 3.5.1 [57]. The pathways are in the form of three columns. The first column is the name of a pathway, and the other two columns are the coding genes connected in the pathway. In total, 151,893 gene pairs (i.e., edges) were obtained as the edge set of pathways.

### 2.2. Overview of the iPS Approach

**Figure 1:**
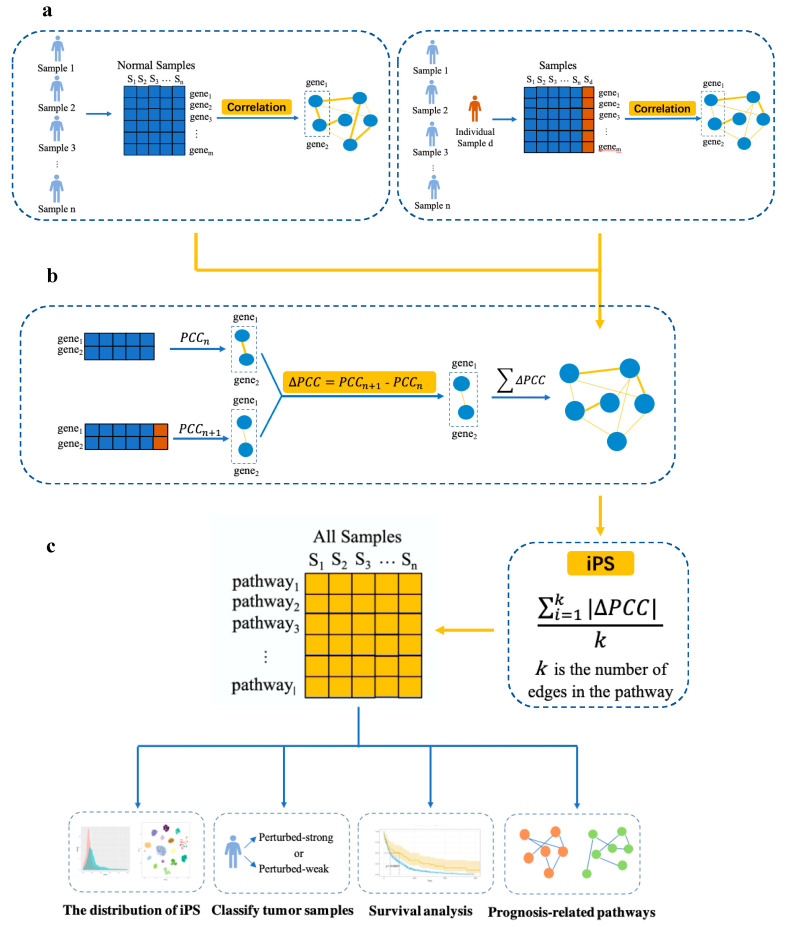
Flowchart of the individual-level pathway score (iPS) approach. (**a**) For a group of n normal samples, compute the correlations, i.e., the $PCC_{n}$ (the Pearson correlation coefficient (PCC) of an edge in each pathway with *n* samples) of each edge in the pathway based on expression data. Then, a new individual sample d is added to the group, and the new correlation of each edge in the pathways is calculated in the same way by the $PCC_{n+1}$ of the combined data. (**b**) The pathway-based differential correlation is calculated by the difference of the corresponding edge in terms of PCC, i.e., ∆PCC = $PCC_{n+1}$ − $PCC_{n}$ for each edge. The sum of all edges, the ∆PCC value, is obtained for each pathway. (**c**) iPS is defined as an individual pathway-based score that can quantify the correlation perturbation of each pathway in each sample. Finally, analyses of distribution, classification, survival, and prognosis are performed.

We first provide an overview as Figure 1 of the pipeline steps. In the first step, individual-level pathways were constructed by using the construction method of the sample-specific network [50]. Second, the individual-level pathway scores (iPSs) of each pathway were calculated for each tumor sample. All the tumor samples were divided into two groups according to the iPS. Finally, prognosis-related pathways were identified for each cancer type by survival analysis.

### 2.3. Construction of Individual-Level Pathways

The normalized gene expression data of a group of normal samples from TCGA served as the reference samples. As shown in Figure 1, we computed the normal samples’ PCC of each pair of genes in an edge as a reference individual-level pathway ($PCC_{n})$. We then added a single sample to the corresponding edge to construct a new individual-level pathway by $PCC_{n+1}$ (the PCC of an edge with a new sample). For this, we can obtain the differential PCC (∆PCC) between the individual-level pathway of all normal samples and the new individual-level pathway of adding a single sample; i.e., ∆PCC measures the correlation perturbation of the edge references to the normal samples. For each sample, we added the absolute value of all ∆PCC in one pathway to construct the individual-level pathways.

### 2.4. Calculation of iPSs

To identify pathways that show unusual patterns at the single sample level, we defined a novel statistic, namely, the individual-level pathway score (iPS). In fact, the iPS measures the average perturbation of all edges in a pathway and is defined as follows, where m is the total number of edges in a pathway:

(1) $$\mathrm{iPS}=\;\frac{\sum_{i=1}^{m}\left|PCC_{n+1}-PCC_{n}\right|}{m},$$

Based on the above process, the iPSs of all samples for each pathway can be calculated. For each pathway, we calculated the mean and standard deviation of all normal samples’ iPSs for each cancer type. If the absolute value of a tumor sample’s iPS was more than two standard deviations away from the normal samples’ mean, this tumor sample was labelled ‘perturbed-strong’ on this pathway, and this pathway was labelled “perturbed-strong” on this sample correspondingly. Otherwise, the tumor sample was labelled ‘perturbed-weak’ on the pathway, and this pathway was labelled “perturbed-weak” on this sample correspondingly. Therefore, for a fixed pathway, there are two groups of “perturbed-strong” and “perturbed-weak” cancer samples with different perturbation degrees on the pathway deviated from that of a healthy, normal level. For a fixed cancer sample, “perturbed-strong” and “perturbed-weak” pathways indicate two groups of pathways with different perturbation degrees on the cancer sample.

### 2.5. Identification of Prognosis-Related Pathways

The prognosis-related pathways were identified through two steps. Firstly, the Wilcoxon test was used to identify differential pathways between perturbed-strong samples and perturbed-week samples by setting FDR < 0.05. Then these differential pathways were used for Kaplan-Meier survival analysis. And a differential pathway was defined as a prognosis-related pathway if perturbed-strong samples had worse prognosis than perturbed-week samples for a given cancer type with a significant log-rank *p*-value < 0.005. We used the “survival” package and “survminer” package in R/Bioconductor to implement the above analysis.

## 3. Results

### 3.1. The Heterogeneity of iPSs

**Figure 2:**
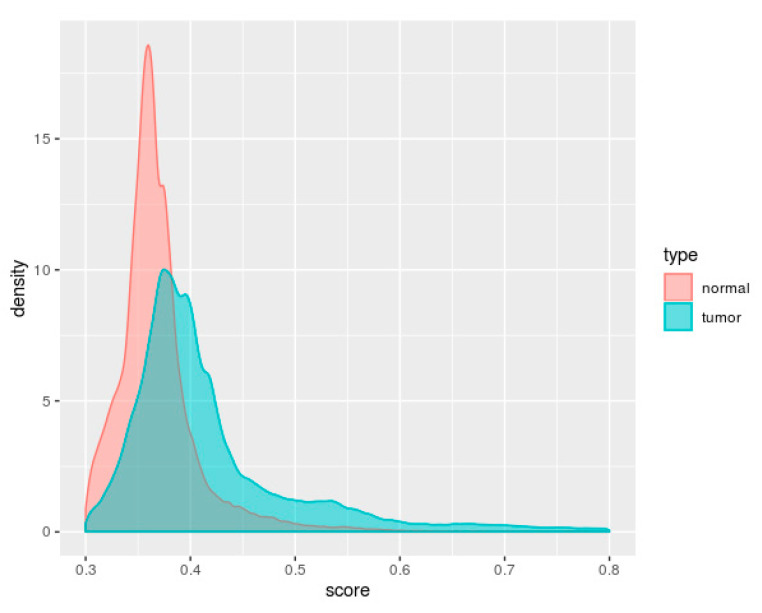
Density plot of the iPS score. The red and green colors represent normal samples and tumor samples, respectively. The iPSs of tumor samples are higher than those of normal samples. Additionally, the tumor samples’ iPSs are more dispersed than those of the normal samples.

For each pathway, the iPSs of all samples (6548 tumor samples and 688 normal samples) were calculated. The distribution of the iPSs in the tumor samples and normal samples is shown in Figure 2. The iPSs of the tumor samples were significantly higher than those of the normal samples, which indicated that the perturbation of pathways in the tumor samples was more serious. In addition, the iPSs of the tumor samples were more dispersed than those of the normal samples.

**Figure 3:**
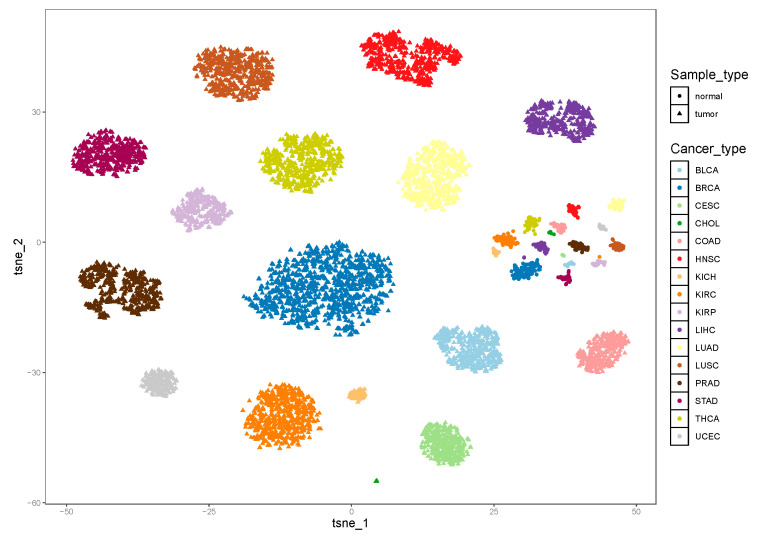
Results of the pan-cancer classification distributed in the *t*SNE plot. Different colors represent different cancer types and corresponding normal samples. The tringles and dots are used to distinguish tumor and normal samples. The number of clusters is 32, which contains 16 cancer types and their corresponding normal types.

Then, we investigated whether iPS could reflect the heterogeneity among different cancer types. By using *t*-distributed stochastic neighbour embedding (*t*-SNE) plots for the tumor and normal samples of 16 cancer types (Figure 3), we observed that samples from the same tumor type (dots with the same color) were obviously clustered together, and different types of normal samples tended to cluster together. In addition, we found that different tumor types (dots with different colors) were separated from each other, and even different types of normal samples could also be separated well. As shown in Figure 3, different normal samples clustered closer than tumor samples, which means that the scores of normal samples were similar, which was in accordance with the result of Figure 2.

**Figure 4:**
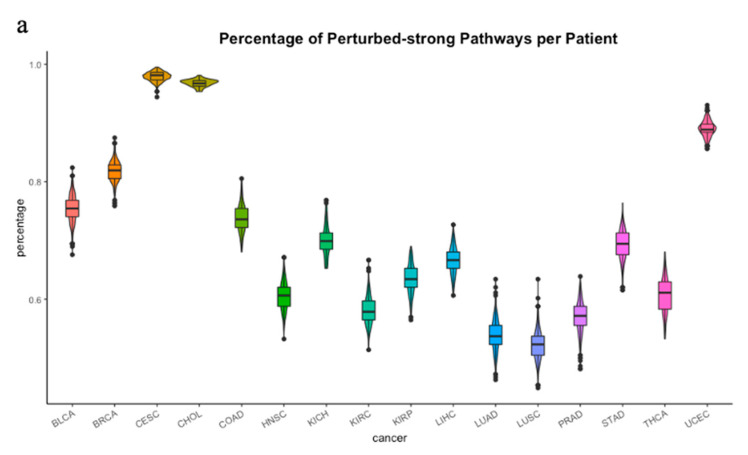

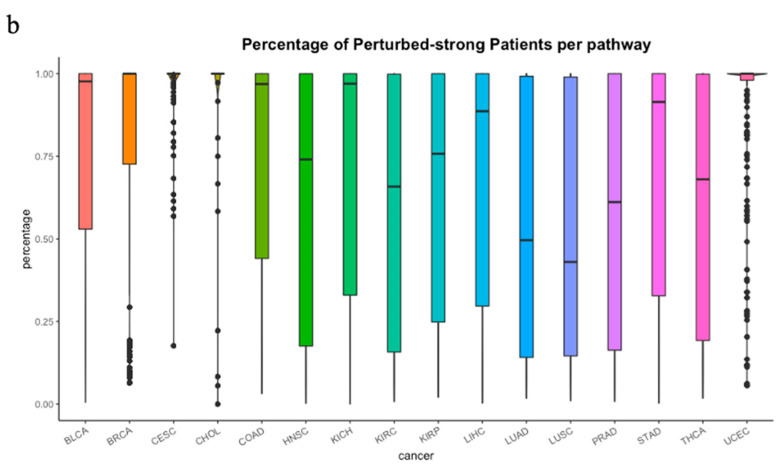
The violin plot of perturbations in each cancer. (**a**) The percentage of perturbed-strong pathways per patient in 16 cancer types. Different colors represent different patient samples of each cancer type. Cervical squamous cell cancer (CESC) and cholangio cancer (CHOL) showed 97% and 96% perturbed pathways, respectively. Breast cancer (BRCA) and uterine corpus endometrial cancer (UCEC) also had high proportions, which were both over 80%. (**b**) The percentage of perturbed-strong patient per pathway. High percentages were observed in CESC, CHOL and UCEC, which were near 100%. The percentages in LUAD and LUSC were below 50%.

For each pathway, each tumor sample was classified as either perturbed-strong or perturbed-weak based on the iPSs. We wanted to know how many pathways were perturbed in a single sample for each cancer type. As shown in Figure 4a, there were remarkable differences among the 16 tumor types in terms of the percentage of perturbed pathways per patient. Cervical squamous cell cancer (CESC) and cholangio cancer (CHOL) showed high proportions of perturbed pathways, which were 97% and 96%, respectively. Because the normal samples’ numbers of CESC and CHOL were less than 20, some results may not have representativeness. Breast cancer (BRCA) and uterine corpus endometrial cancer (UCEC) also had high proportions, which were both over 80%. Frequent malignancy in women may induce strong perturbations in samples [58,59]. Conversely, lung cancer (LUSC) showed the lowest proportions (53%). For the 16 cancer types, the perturbation proportions of the tumor samples in each pathway (Figure 4b) were obviously different. Cervical squamous cell cancer (CESC), cholangio cancer (CHOL), breast cancer (BRCA), and uterine corpus endometrial cancer (UCEC) showed higher percentages than the other cancer types.

### 3.2. Prognosis-Related Pathway

In total, 76 prognostic pathways in a range of 1 (in cervical squamous cell cancer (CESC), colon cancer (COAD), kidney chromophobe cancer (KICH), kidney renal papillary cell cancer (KIRP), and uterine corpus endometrial cancer (UCEC) to 14 breast cancer (BRCA) cancer types were obtained among a total of 216 pathways and 16 cancer types (Table S1).

By counting the number of significant prognosis-related pathways in different cancer types, we found that the ratios of prognosis-related pathways were imbalanced among the 16 different types of cancer. In breast cancer (BRCA), 14 pathways were found, which had significant differences between perturbed-strong and perturbed-weak patients. In head and neck squamous cell cancer (HNSC) and stomach cancer (STAD), we obtained 13 and 12 prognosis-related pathways, respectively. We also found that the number of shared prognosis-related pathways in all cancer types was very small (Table S2), which indicates strong heterogeneity among different cancer types. Thus, the majority of prognosis-related pathways are cancer-type-specific.

**Figure 5:**
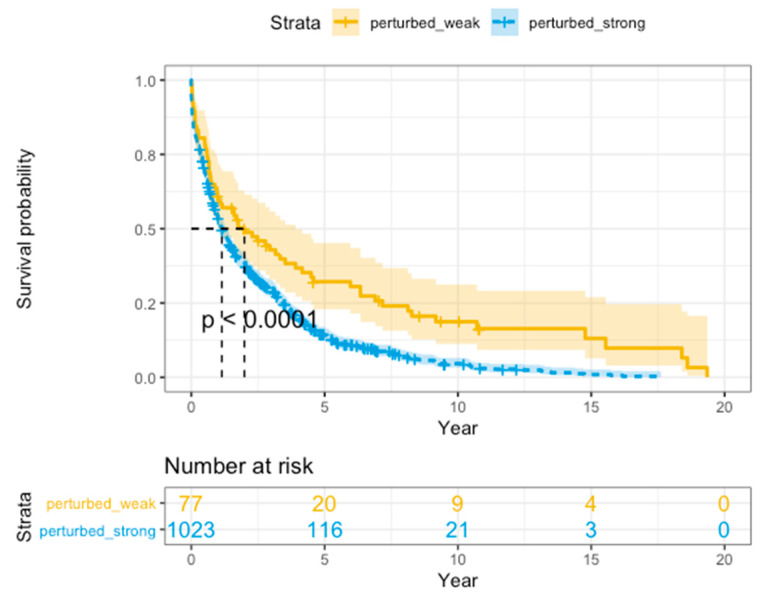
Kaplan–Meier survival analysis. The survival curves of the perturbed-weak and perturbed-strong groups of BRCA based on the iPS in the DAP12 interaction pathway. The *X* axis is survival in days. The *Y* axis is the overall survival rate.

Breast cancer is one of the most common malignant tumors in women. According to previous reports, multiple pathways have been proven to play an important role in the tumorigenesis and progression of breast cancer [60,61,62,63,64,65]. We found some prognosis-related pathways of breast cancer, such as the regulation of insulin-like growth factor (IGF) transport and uptake by insulin-like growth factor binding proteins (IGFBPs), the PI3K/Akt signal transduction pathway, signaling by PDGF, Toll-like receptor cascades, DAP12 interactions, and constitutive signaling by aberrant PI3K in cancer. The regulation of IGF transport and uptake by IGFBPs has been proven to be associated with the occurrence of breast cancer by activating the EGFR and IGFR signaling pathways [66,67,68,69]. IGFR-1 is thought to be related to the malignant transformation of breast cancer cells in vivo and in vitro [66]. Dong et al. showed that IGF-1-mediated MAPK and PI3K/Akt signal transduction pathways were abnormally activated in the breast cancer cell line MCF-7 [67]. Worster et al. showed that IGF-I and EGF cooperated with each other to affect cell proliferation through the Akt and ERK signaling pathways [68]. For the DAP12 interaction pathway, Shabo et al. studied the relationship between DAP12 expression in breast cancer cells and disease progression and found that breast cancer patients with high DAP12 expression had poor prognosis, high recurrence rates and short survival times due to liver metastasis [69] (Figure 5).

There were also some verified prognosis-related pathways identified by iPS in pan-cancer. In head and neck squamous cell cancer (HNSC), signaling by NOTCH4 was identified (Figure S1). NOTCH4 was reported to inhibit NOTCH1 signaling in cis by binding to NOTCH1 and interfering with the S1 cleavage of NOTCH1, thus preventing the production of functional NOTCH1 heterodimers at the cell surface. Research suggests that the mutation frequency of Notch 1 in HNSC is approximately 22%, which is similar to that of the TP53 gene [70]. In stomach cancer (STAD), constitutive signaling by aberrant PI3K in cancer, PTEN loss of function in cancer (Figure S2a), deadenylation-dependent mRNA decay (Figure S2b), and transcriptional regulation of pluripotent stem cells (Figure S2c) are pathways that are well known to play important roles in the tumorigenesis and development of STAD [71,72]. The survival outcome results were consistent with those reported. Additionally, breast cancer has four major subtypes: basal-like, HER2+, luminal A, and luminal B, which have substantial pathological differences. We tried to apply the iPS to the four breast cancer (BRCA) subtypes separately to explore whether the disruption of pathways occurs in the subtypes of breast cancer, and 2, 1, 7, and 1 significant biomarker pathways were identified in the basal-like, HER2+, luminal A, and luminal B subtypes, respectively (Table S3).

### 3.3. Genes in Prognosis-Related Pathways

Crucial oncogenes have been implicated in many cancer types [73]. As reported, 127 genes have been proven to have significant effects on patient survival in 11 cancer types [74]. In our study, with the identification of prognosis-related pathways using iPS, we counted the degree of genes in the relevant prognostic pathways by Cytoscape. We found that most important cancer driver genes had high degrees and were also associated with patient survival in different cancer types. We observed that KRAS, PIK3R1, PIK3CA, CDKN1B, PTEN, and NF1 showed high degrees (Table S4) in the PI3K/Akt signal transduction pathway. Experiments have confirmed that PTEN mainly acts on the downstream target gene PIP3 of PI3K, thereby blocking the PI3K/AKT signaling pathway to inhibit cancer development [75,76]. Kandoth et al. showed that mutations in CDKN1B had effects on the survival of breast cancer (BRCA) patients [74]. Griffith et al. found that somatic mutations in NF1 and PIK3R1 had adverse effects on the prognosis of BRCA [77].

We also observed that most genes in the NOTCH signaling pathway, such as NOTCH1, NOTCH3, RBPJ, EP300, and TBL1XR1, had high degrees (Table S4). It has been proven that the Notch receptor and ligand have abnormal expression in various types of lung cancer, and the transcription factor RBPJ is a component of the Notch signaling pathway [78]. Under normal oxygen concentrations, the proliferation of lung adenocarcinoma cells can be inhibited by blocking the Notch1 signaling pathway [79,80]. Zheng et al. showed that Notch3 can act as an oncogene in lung cancer [81,82]. In addition, the TBLIXRI gene was significantly highly expressed in LUSC [82].

In the Toll-like receptor signaling pathway, related adaptor proteins are associated with the occurrence, development, metastasis, and invasion of breast cancer (BRCA). From Table S4, TLR4 had the highest degree. Mehmeti et al. proved that TLR4 was expressed in a functional form in oestrogen receptor (ER)/progesterone receptor (PR)-negative breast cancers, and a gene expression analysis of primary breast cancers showed a strong correlation between TLR4 expression and the expression of pro-inflammatory mediators [83]. Ma et al. confirmed that TLR4-MyD88/MyD88 is a risk factor for the prognosis of breast cancer (BRCA) [84]. Volk Draper et al. revealed that intervention with anti-TLR4 during or after chemotherapy can inhibit the inflammatory pathway of tumor progression [85]. Therefore, TLR4 has a negative impact on the prognosis of breast cancer patients, and its expression may improve the prognosis of breast cancer patients. Additionally, CD14, TRAF6, and IRAK1 had higher degrees than other genes in the Toll-like receptor signaling pathway (Table S4). The inhibition of TLR2 or its downstream targets CD14, MyD88, and IRAK1 can inhibit the proliferation of human breast cancer (BRCA) cells [86]. The abnormal expression of TRAF6 is closely related to carcinogenesis by participating in the regulation of tumor cell apoptosis, growth, and invasion through different signaling pathways [87].

### 3.4. Comparison with GSVA and ssGSEA

In our study, the iPS method was able to identify the perturbation degree of individual tumor sample on pathway levels by calculating iPS for each sample per pathway. Gene Set Variation Analysis (GSVA) [88] is a non-parametric, unsupervised method for estimating variation of gene set enrichment through the samples of an expression data set. GSVA performs a change in coordinate systems, transforming the expression matrix of genes into the expression matrix of gene sets, thereby allowing the evaluation of pathway enrichment for each sample. Single sample GSEA (i.e., ssGSEA) [89] is another approach to identify abnormal pathways at the individual sample level. Thus, both GSVE and ssGESA are capable of producing a summary statistic for every pathway in individual level. In order to compare these three methods, we used the empirical Bayes test (R ‘limma’ package) to identify significantly differential pathways between tumor and normal samples.

Supplementary Tables S5 and S6 list the top ten significant pathways identified by the three methods in breast cancer (BRCA) and lung cancer (LUAD). For BRCA, four pathways identified by the iPS were cancer-related (red), while three in the GSVA and ssGSEA lists were cancer-related (red), respectively. Six, four and four pathways in the iPS, GSVA, and ssGSEA lists in LUAD were cancer-related (red), respectively. In addition, these cancer-related pathways identified by iPS and other two methods almost have no overlap.

## 4. Discussion

Studies of pathway-based approaches facilitate the identification of robust prognosis-related features. Compared with traditional single-molecule biomarkers, pathway-based biomarkers more accurately characterize disease states because more information on interactions and networks is considered [31]. In our study, we did not use the predefined gene sets of pathways but instead used the gene interactions in the pathway (i.e., the gene pairs in the pathways). Here, we describe the individual-level pathway score (iPS), a computational method that identifies prognosis-related pathways by defining each sample as perturbed-strong or perturbed-weak in different cancer types. Based on our results, we concluded that iPS can measure a patient’s clinical features and can be useful for identifying individual samples of different cancer types, including normal and tumor samples, by expression data. The core of iPS is to calculate a score per pathway, which may contain hundreds of genes per individual sample, and to measure the deviation level of the pathway from that in the normal samples. We found some prognosis-related pathways for most of the cancer types, and these biomarkers had an advantage in survival risk stratification. Our method generates clinically relevant stratifications, and outcome predictors and pathways include more accurate information than single genes in terms of predicting clinical outcomes. In the breast cancer (BRCA) and REACTOME pathways, PIP3 activates AKT signaling and was identified by iPS as a prognosis-related pathway. The abnormal activation of this pathway is closely related to the occurrence and development of breast cancer. The regulation of insulin-like growth factor (IGF) transport and uptake by insulin-like growth factor binding proteins (IGFBPs) was also identified as a prognosis-related pathway. Additionally, the majority of pathway-based biomarkers are perturbed in most fractions of all patients. Among the two given cancer types, their pathway-based biomarkers had little overlap. These results confirmed the fact that cancer is a highly heterogeneous disease, so personalized treatment is necessary for patients with different cancer types. Thus, our findings warrant further research.

Though we have developed a statistical method for the classification and identification of prognosis-related pathways in different cancer types, there are a few limitations in our study. First, we only used the REACTOME database and filtered 216 pathways, which did not contain more information from different databases. Second, using iPS to classify survival subtypes in BRCA, we did not obtain ideal stratification results. Third, we only tested a single pathway to examine whether a given pathway is predictive of a clinical outcome. The prediction performance of the cooperation of multiple pathways may produce better results.

In summary, iPS can be applied extensively to many types of cancer as long as the given expression data of individual samples and corresponding normal samples can be used to measure the perturbed level of each pathway between tumor samples and normal samples. Thus, iPS can estimate the large-scale changes that occur in a small minority of patients, even in a single patient. For prediction purposes, we also expect that our method is applicable to more cancer types with good performance. Validation with other large size data is also desired for future research. Also, the iPS scores only consider the perturbation degrees of an individual cancer sample on pathways references to normal samples. The perturbation directions (i.e., the positive and negative differential correlations), which convey different information, should be considered further. This represents one potential future research direction for the continuous development of iPS. Our findings represent potential future research directions.

## 5. Conclusions

In the current study, iPS was used to identify prognosis-related pathways at the individual level. A comparison analysis was performed between tumor samples and normal samples in 16 cancer types. We calculated the degree of iPS deviation from that of normal samples and demonstrated that iPS can sensitively capture relevant biological and clinical information. Our results showed that iPS can distinguish different cancer types well. The prognosis-related pathways of the 16 cancer types have excellent ability to distinguish different perturbed degree of samples. We trust that our pathway-based method is reliable in prognostic prediction, especially for precision medicine.

## Figures and Tables

**Table 1:** Cancer types and the number of samples used from The Cancer Genome Atlas (TCGA).

Cancer Type	Normal Samples	Tumor Samples
Bladder urothelial carcinoma (BLCA)	19	408
Breast invasive carcinoma (BRCA)	113	1102
Cervical squamous cell carcinoma and endocervical adenocarcinoma (CESC)	3	306
Cholangiocarcinoma (CHOL)	9	36
Colon adenocarcinoma (COAD)	41	287
Kidney chromophobe (KICH)	25	66
Kidney renal clear cell carcinoma (KIRC)	72	534
Kidney renal papillary cell carcinoma (KIRP)	32	291
Liver hepatocellular carcinoma (LIHC)	50	374
Lung adenocarcinoma (LUAD)	59	517
Lung squamous cell carcinoma (LUSC)	51	502
Prostate adenocarcinoma (PRAD)	52	498
Stomach adenocarcinoma (STAD)	35	415
Head and neck squamous cell carcinoma (HNSC)	44	522
Thyroid carcinoma (THCA)	59	513
Uterine corpus endometrial carcinoma (UCEC)	24	177
Total	688	6548

## Supplementary Materials

The following are available online at https://www.mdpi.com/2073-4425/11/11/1281/s1, Figure S1: Significant survival differences for the mutation frequency of Notch4 by iPS in HNSC. Figure S2: Identification of significant survival differences for prognostic biomarker pathways by iPS in STAD. (a) Constitutive Signaling by Aberrant PI3K in Cancer and PTEN Loss of Function in Cancer, (b) Deadenylation-dependent mRNA decay, (c) Transcriptional regulation of pluripotent stem cells pathways. Table S1: Identification of 76 prognostic biomarker pathways in 16 cancer types. Table S2: The number of same prognostic biomarker pathways among 16 cancer type. Table S3: Identification of prognostic biomarker pathways in breast cancer subtypes. Table S4: The degree of genes in the relevant prognostic pathways. Table S5: Top ten pathways differentiated between tumor and normal by iPS, GSVA and ssGSEA in BRCA. Table S6: Top ten pathways differentiated between tumor and normal by iPS, GSVA and ssGSEA in LUAD.
