# Supplementary material for: Revealing Prognosis-Related Pathways at the Individual Level by a Comprehensive Analysis of Different Cancer Transcription Data
Source: Genes (Basel). 2020 Oct 29;11(11):1281. doi: 10.3390/genes11111281 (PMC7692404; doi:10.3390/genes11111281)
Supplement: Supplementary file 1 [file genes-11-01281-s001.pdf]

genes MDPI

Table 1. Identification of 76 prognostic biomarker pathways in 16 cancer types.

REACTOME Pathways	Edges number	Cancer Type
Oxidative Stress Induced Senescence	7923	BLCA
Fc epsilon receptor (FCERI) signaling	2898	
Transcriptional regulation of white adipocyte differentiation	1933	
mRNA Capping	380	
RIG-I. MDA5 mediated induction of IFN-alpha	319	
PIP3 activates AKT signaling, Constitutive Signaling by Aberrant PI3K in Cancer, Constitutive Signaling by AKT1 E17K in Cancer, and PTEN Loss of Function in Cancer	8067	BRCA
Signaling by ROBO receptors	7342	
Signaling by Rho GTPases	6566	
Nucleotide Excision Repair	3011	
Signaling by PDGF	2918	
DAP12 interactions	2645	
Senescence-Associated Secretory Phenotype (SASP)	2601	
Pre-NOTCH Expression and Processing and Defective LFNG causes SCDO3	1381	
Toll-Like Receptors Cascades	803	
Protein folding	649	
tRNA processing in the nucleus	552	
Transcriptional regulation of pluripotent stem cells	342	
RIG-I. MDA5 mediated induction of IFN-alpha	319	
Regulation of Insulin-like Growth Factor (IGF) transport and uptake by Insulin-like Growth Factor Binding Proteins (IGFBPs)	259	
Processing of Capped Intron-Containing Pre-mRNA	17505	CESC
Resolution of Abasic Sites (AP sites)	155	COAD
SRP-dependent cotranslational protein targeting to membrane	5212	HNSC
Metabolism of amino acids and derivatives	4563	
SCF-KIT signaling	2875	
Signaling by VEGF	2014	
Estrogen responsive signaling and Estrogen-responsive signaling in cancer	1879	
Regulation of Hypoxia-inducible Factor (HIF) by oxygen	1828	
Synthesis of DNA	1821	
Signaling by NOTCH4	968	
Fcgamma receptor (FCGR) dependent phagocytosis	917	
Collagen biosynthesis and modifying enzymes and Disorders of collagen biosynthesis and modifying enzymes	306	
Cell surface interactions at the vascular wall	154	
Signaling by BMP	105	
Nephrin family interactions	57	
Signaling by NOTCH2	411	KICH
Mitochondrial translation	4165	KIRC
Clathrin-mediated endocytosis	3978	
Signaling by NOTCH4	968	
Platelet Aggregation (Plug Formation)	202	KIRP
PIP3 activates AKT signaling, Constitutive Signaling by Aberrant PI3K in Cancer, Constitutive Signaling by AKT1 E17K in Cancer, and PTEN Loss of Function in Cancer	8067	LIHC
Mitochondrial translation	4165	

Hedgehog 'off' state	1493	
Costimulation by the CD28 family	484	
Netrin-1 signaling	148	
Telomere Maintenance	139	
Nephrin family interactions	57	
Class I MHC mediated antigen processing & presentation	31068	LUAD
Inositol phosphate metabolism	660	
DNA Damage Bypass	635	
Mitochondrial protein import	135	
Prolactin receptor signaling	76	
RORA activates gene expression	75	
GP1b-IX-V activation signaling	25	
Collagen degradation	23	
Post-translational protein modification	11613	LUSC
Signaling by NOTCH2	411	
Formation of Fibrin Clot (Clotting Cascade)	154	
Metabolism of lipids	1982	PRAD
Estrogen responsive signaling and Estrogen-responsive signaling in cancer	1879	
Cytosolic sensors of pathogen-associated	572	
PIP3 activates AKT signaling, Constitutive Signaling by Aberrant PI3K in Cancer, Constitutive Signaling by AKT1 E17K in Cancer, and PTEN Loss of Function in Cancer	8067	STAD
Nonsense-Mediated Decay (NMD)	5775	
Regulation of mitotic cell cycle	3085	
Nonhomologous End-Joining (NHEJ)	1722	
Pre-NOTCH Expression and Processing and Defective LFNG causes SCDO3	1381	
Interferon Signaling	1135	
DNA Damage Bypass	635	
Deadenylation-dependent mRNA decay	416	
Transcriptional regulation of pluripotent stem cells	342	
Prolactin receptor signaling	76	
Incretin synthesis secretion and inactivation	68	
Binding and Uptake of Ligands by Scavenger Receptors	48	
Fanconi Anemia Pathway	5143	THCA
TCR signaling	1772	
Nucleosome assembly	351	
NCAM signaling for neurite out-growth	1763	UCEC

Table 2. The number of same prognostic biomarker pathways among 16 cancer type.

REACTOME Pathways	Edges number	Cancer Type
RIG-I. MDA5 mediated induction of IFN-alpha	319	BLCA<br>BRCA
PIP3 activates AKT signaling, Constitutive Signaling by Aberrant PI3K in Cancer, Constitutive Signaling by AKT1 E17K in Cancer, and PTEN Loss of Function in Cancer	8067	BRCA<br>LIHC<br>STAD
Transcriptional regulation of pluripotent stem cells	342	BRCA<br>STAD
Estrogen responsive signaling and Estrogen-responsive signaling in cancer	1879	HNSC<br>PRAD
Signaling by NOTCH4	968	HNSC<br>KIRC
Nephrin family interactions	57	HNSC<br>LIHC
Signaling by NOTCH2	411	KICH<br>LUSC

Mitochondrial translation	4165	KIRC LIHC
DNA Damage Bypass	635	LUAD STAD
Prolactin receptor signaling	76	LUAD STAD

Table 3. Identification of prognostic biomarker pathways in breast cancer subtypes.

REACTOME Pathways	Edges number	Subtypes
PIP3 activates AKT signaling, Constitutive Signaling by Aberrant PI3K in Cancer, Constitutive Signaling by AKT1 E17K in Cancer, and PTEN Loss of Function in Cancer<br>Signaling by ROBO receptors	2	Basal like
Signaling by ROBO receptors	1	HER2+
DAP12 interactions<br>Senescence-Associated Secretory Phenotype (SASP)<br>Toll-Like Receptors<br>Protein folding<br>tRNA processing in the nucleus<br>RIG-I.MDA5 mediated induction of IFN-alpha.betaedge<br>Regulation of Insulin-like Growth Factor (IGF) transport and uptake by Insulin-like Growth Factor Binding Proteins (IGFBPs)	7	Luminal A
PIP3 activates AKT signaling, Constitutive Signaling by Aberrant PI3K in Cancer, Constitutive Signaling by AKT1 E17K in Cancer, and PTEN Loss of Function in Cancer	1	Luminal B

Table 4. The degree of genes in the relevant prognostic pathways.PI3K/Akt signal transduction pathway (degree > 50).

Gene	Degree	Gene	Degree	Gene	Degree
KRAS	152	PTPN11	99	MYD88	77
HRAS	152	CDK2	98	IL1RL1	77
NRAS	152	TRAF6	88	IL33	77
RPS27A	148	PIK3R1	86	SOS1	74
UBA52	148	PIK3CA	82	CDKN1B	71
UBC	148	IRAK4	77	CDKN1A	70
UBB	148	IL1RAP	77	PTEN	67
GRB2	125	IRAK1	77	NF1	56

NOTCH2 signaling pathway (degree >= 10).

Gene	Degree	Gene	Degree	Gene	Degree
NOTCH1	67	CREBBP	17	TFDP1	12
RBPJ	29	KAT2B	17	TFDP2	12
SNW1	27	UBC	17	NCOR1	11
NOTCH2	26	UBA52	17	NCOR2	11
EP300	19	DTX1	16	ELF3	11
MAML1	19	CCND1	13	TBL1X	11
MAML3	19	E2F1	12	TBL1XR1	11
MAML2	19	E2F3	12	CDK8	10
RPS27A	17	NOTCH3	12	CCNC	10

Toll-Like Receptors pathway (degree >= 20)

Gene	Degree	Gene	Degree	Gene	Degree
TLR4	61	MYD88	35	RIPK1	25
LY96	58	IRAK2	32	IRAK4	22

CD14	58	IKBKG	32	TLR9	22
TRAF6	47	IRAK1	31	IRF7	21
TLR3	37	CHUK	28	NFKB1	20
TICAM2	37	IKBKB	28		
TICAM1	36	UBE2N	26		

Table 5. Top ten pathways differentiated between tumor and normal by iPS, GSVA and ssGSEA in BRCA.

Pathways identified by iPS	P-value	Adj P-value
Mitochondrial translation	0	0
Reactive oxygen species (ROS)	0	0
trans-Golgi Network Vesicle Budding	0	0
Endosomal Sorting Complex Required For Transport (ESCRT)	0	0
Potassium Channels	0	0
FI Nework for Aquaporin-mediated transport	0	0
HSP90 chaperone cycle for steroid hormone receptors (SHR)	0	0
Metabolism of nitric oxide	0	0
mRNA Capping	0	0
COPI-mediated anterograde transport	0	0

Pathways identified by GSVA	P-value	Adj P-value
Peroxisomal protein import	2.74E-27	6.10E-25
Cytosolic sensors of pathogen-associated	1.09E-25	1.21E-23
Class I MHC mediated antigen processing & presentation	1.56E-23	1.16E-21
DNA Double Strand Break Response	2.38E-22	7.58E-21
Fanconi Anemia Pathway	2.38E-22	7.58E-21
Homology Directed Repair	2.38E-22	7.58E-21
Nonhomologous End-Joining (NHEJ)	2.38E-22	7.58E-21
Cilium Assembly	1.01E-21	2.82E-20
Prolactin receptor signaling	2.82E-21	6.98E-20
Integration of energy metabolism and Defective ABCC8 can cause hypoglycemias and hyperglycemias	6.72E-19	1.50E-17

Pathways identified by ssGSEA	P-value	Adj P-value
DNA Double Strand Break Response	1.02E-33	5.67E-32
Fanconi Anemia Pathway	1.02E-33	5.67E-32
Homology Directed Repair	1.02E-33	5.67E-32
Nonhomologous End-Joining (NHEJ)	1.02E-33	5.67E-32
Peroxisomal protein import	2.35E-33	1.05E-31
Cytosolic sensors of pathogen-associated	4.27E-33	1.59E-31
Prolactin receptor signaling	1.93E-30	6.16E-29
Integration of energy metabolism and Defective ABCC8 can cause hypoglycemias and hyperglycemias	2.71E-30	7.57E-29
Nucleosome assembly	4.63E-29	1.15E-27
Class I MHC mediated antigen processing & presentation	5.68E-28	1.27E-26

Table 6. Top ten pathways differentiated between tumor and normal by iPS, GSVA and ssGSEA in LUAD.

Pathways identified by iPS	P-value	Adj P-value
Signaling by NTRK3 (TRKC)	2.93E-17	6.34E-15
Estrogen responsive signaling and Estrogen-responsive signaling in cancer	4.51E-16	4.87E-14
Transmission across Chemical Synapses	1.43E-15	1.03E-13
TCR signaling	8.19E-15	4.42E-13
Formation of Fibrin Clot (Clotting Cascade)	4.72E-14	2.04E-12

Mitotic Prophase	8.59E-14	2.77E-12
Peroxisomal protein import	8.99E-14	2.77E-12
Signaling by Type 1 Insulin-like Growth Factor 1 Receptor (IGF1R)	3.50E-13	9.46E-12
Signaling by NOTCH4	4.40E-13	1.06E-11
Metabolism of nucleotides	1.83E-12	3.95E-11

**Pathways identified by GSVA**	**P-value**	**Adj P-value**
Activation of anterior HOX genes in hindbrain development during early embryogenesis	6.78E-08	1.51E-05
Hh 'on'	5.48E-07	6.11E-05
Cell surface interactions at the vascular wall	4.52E-06	0.0003363
Plasma lipoprotein assembly, remodeling, and clearance and Defective ABCA1 causes Tangier disease	7.65E-06	0.00042675
Signaling by ERBB4	3.64E-05	0.00135433
Signaling by Hippo	3.64E-05	0.00135433
Class I MHC mediated antigen processing & presentation	6.34E-05	0.00179838
Peptide hormone biosynthesis	6.45E-05	0.00179838
Smooth Muscle Contraction	0.00086716	0.02014773
Cellular response to heat stress	0.00090349	0.02014773

**Pathways identified by ssGSEA**	**P-value**	**Adj P-value**
Activation of anterior HOX genes in hindbrain development during early embryogenesis	7.47E-10	1.67E-07
Cell surface interactions at the vascular wall	3.81E-07	4.25E-05
Hh 'on'	8.09E-07	6.01E-05
Regulation of mitotic cell cycle	1.66E-06	9.26E-05
Class I MHC mediated antigen processing & presentation	3.85E-06	0.0001716
Signaling by ERBB4	6.27E-06	0.0001998
Signaling by Hippo	6.27E-06	0.0001998
Plasma lipoprotein assembly, remodeling, and clearance and Defective ABCA1 causes Tangier disease	9.20E-06	0.00025658
Smooth Muscle Contraction	7.19E-05	0.00178042
Response to elevated platelet cytosolic Ca2+	0.00015407	0.00343576

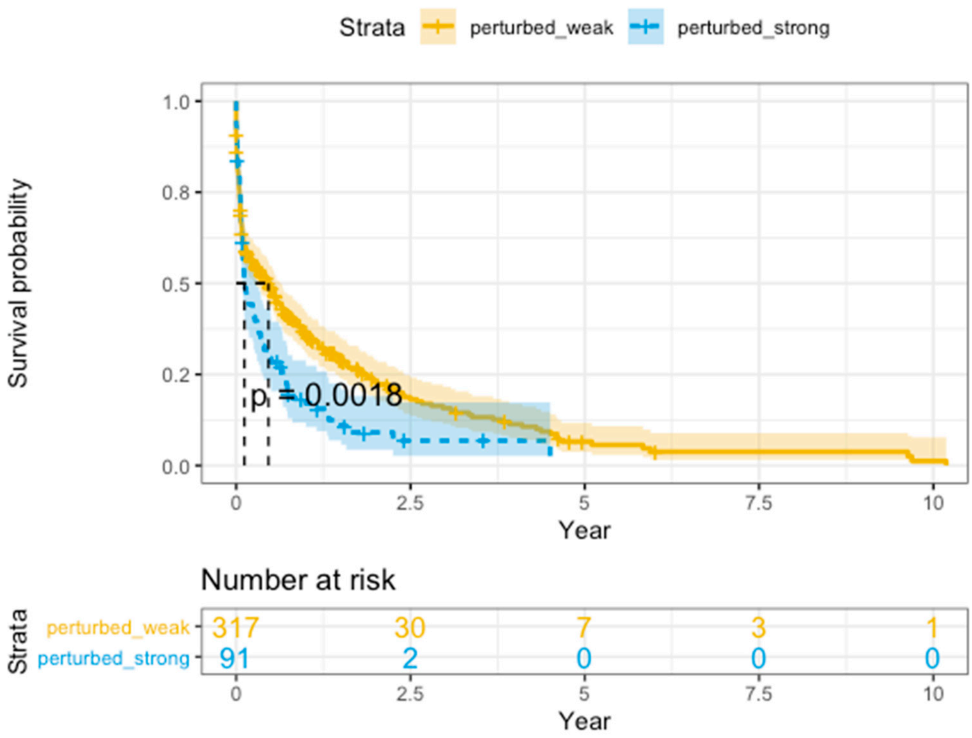

Figure 1. Significant survival differences for the mutation frequency of Notch4 by iPS in HNSC.

a

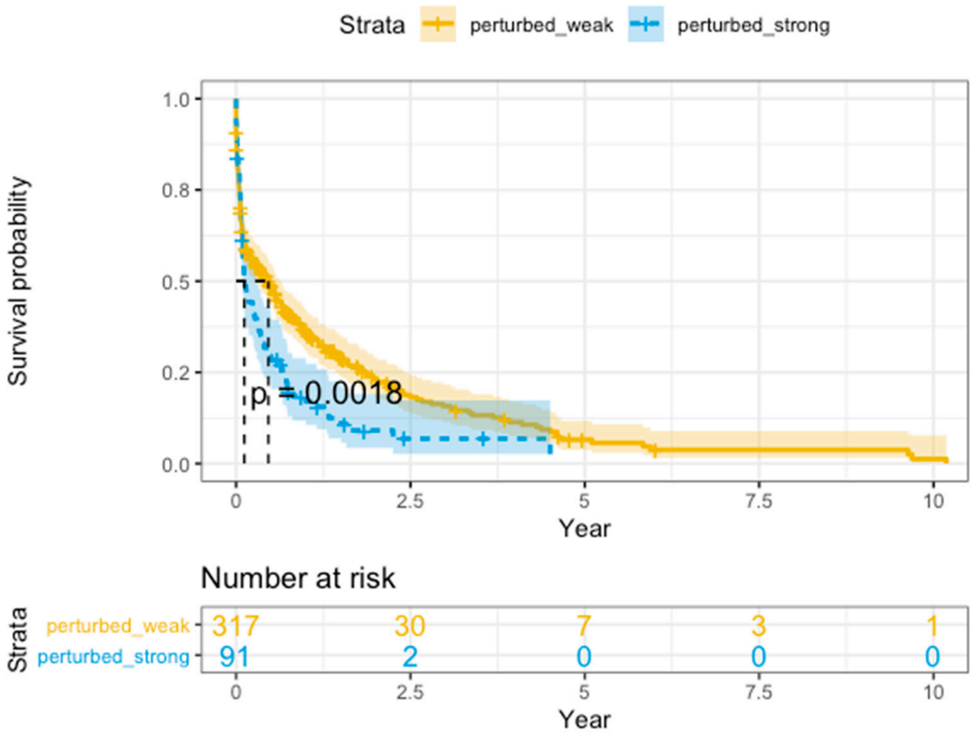

b

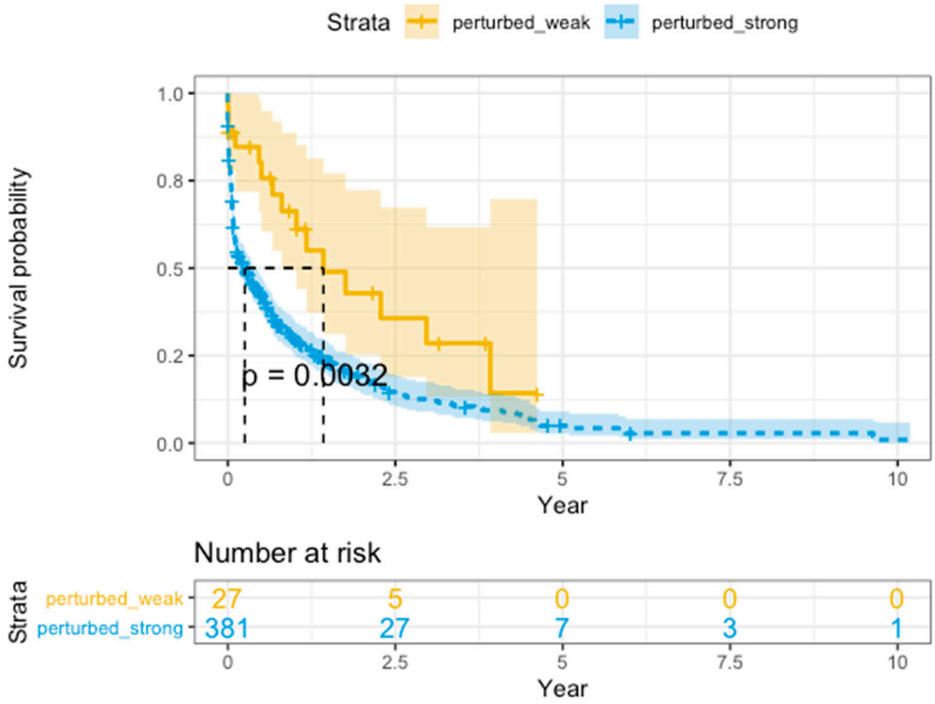

c

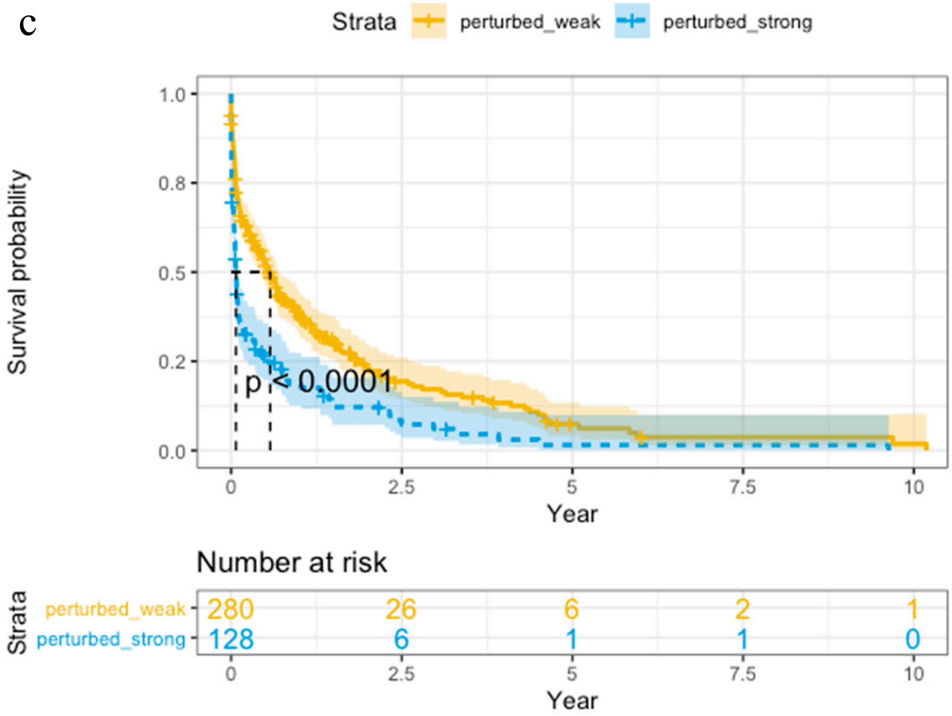

Figure 2. Identification of significant survival differences for prognostic biomarker pathways by iPS in STAD. (a) Constitutive Signaling by Aberrant PI3K in Cancer and PTEN Loss of Function in Cancer, (b) Deadenylation-dependent mRNA decay, (c) Transcriptional regulation of pluripotent stem cells pathways.
